# Pan-immune-inflammation value predicts 2-year recurrence after chemoradiotherapy in nasopharyngeal carcinoma

**DOI:** 10.3389/fmed.2026.1774518

**Published:** 2026-06-24

**Authors:** Ke Du, Zhaoyuan Li, Qi Zhang, Zeng Liu

**Affiliations:** Department of oncology, Xiangyang Central Hospital, Affiliated Hospital of Hubei University of Arts and Science, Xiangcheng District, Xiangyang City, Hubei Province, China

**Keywords:** chemoradiotherapy, concurrent, nasopharyngeal carcinoma, pan-immune-inflammation value, recurrence

## Abstract

**Background:**

Nasopharyngeal carcinoma (NPC) remains a clinically challenging head and neck malignancy with a substantial risk of post-treatment recurrence, underscoring the need for accessible biomarkers that complement established staging and Epstein-Barr virus (EBV)-DNA assessment. This retrospective cohort study evaluated the prognostic utility of the pan-immune-inflammation value (PIV), a composite biomarker integrating neutrophil, platelet, monocyte, and lymphocyte counts, for predicting 2-year recurrence in patients with NPC treated with chemoradiotherapy.

**Methods:**

We retrospectively analyzed 306 patients with NPC treated at a tertiary center between February 2017 and April 2023, with follow-up until recurrence or April 2025. Baseline clinical, laboratory, imaging, and pretreatment EBV-DNA data were evaluated, and patients were stratified into recurrence and recurrence-free groups according to 2-year follow-up. Predictive performance and survival associations were assessed using receiver operating characteristic (ROC) analysis, Kaplan-Meier analysis with the log-rank test, and univariate and multivariate Cox regression models.

**Results:**

PIV was significantly elevated in the recurrence group (380.88 ± 204.99 vs. 329.76 ± 193.92, *P* = 0.037), indicating its association with immune-inflammatory activation. Receiver operating characteristic (ROC) analysis demonstrated moderate predictive accuracy (AUC = 0.582, 95% CI: 0.512–0.651, *P* = 0.023), with an optimal cutoff of 381.55 yielding 44.7% sensitivity and 73.1% specificity. Kaplan-Meier survival analysis revealed shorter recurrence-free survival (RFS) in high-PIV patients (mean RFS: 18.69 ± 0.75 vs. 20.41 ± 0.48 months, log-rank *P* = 0.004). Univariate and multivariate Cox regression confirmed advanced T stage (HR = 2.354, 95% CI: 1.421–3.900, *P* = 0.001) and elevated PIV (HR = 1.698, 95% CI: 1.130–2.553, *P* = 0.011) as independent predictors of recurrence.

**Conclusions:**

These findings suggest that PIV may serve as a cost-effective adjunctive tool for recurrence risk stratification, although the retrospective design and moderate sensitivity indicate that prospective multicenter validation is required before routine clinical application.

## 1 Introduction

Nasopharyngeal carcinoma (NPC) represents an exceptionally malignant epithelial neoplasm that predominantly afflicts individuals of East and Southeast Asian descent, with notably elevated incidence rates noted in regions, involving southern China and certain areas in Southeast Asia (1, 2). Characterized by a distinct array of pathological features and clinical manifestations, NPC is frequently linked to Epstein-Barr virus (EBV) infection, a factor that is hypothesized to play a notable function in the tumorigenesis and progression of the disease (3). Originating in the nasopharyngeal epithelium, a site anatomically deep in the upper respiratory tract, NPC is notoriously difficult to detect in its early stages owing to its nonspecific clinical presentation and lack of overt symptoms (4). This diagnostic challenge mainly results in the detection of NPC at more advanced, locally invasive stages, remarkably complicating both therapeutic intervention and prognostic evaluation. The standard therapeutic approach for NPC typically involves a multimodal regimen that integrates radiation therapy with chemotherapy, with concurrent chemoradiotherapy (CCRT) emerging as the preferred treatment strategy for locally advanced cases (5, 6). CCRT has been demonstrated to noticeably improve survival outcomes by enhancing tumor radiosensitivity and facilitating the concurrent treatment of the primary tumor and regional lymphatic metastases (5, 6). However, despite noticeable advances in therapeutic modalities, NPC's recurrence remains a pervasive and formidable issue, with a notable proportion of cases experiencing relapse within 2 years following the completion of initial therapy (7). This recurrence, particularly when it involves distant metastatic spread, is invariably linked to a remarkably poorer prognosis and presents a noticeable challenge to the long-term management of NPC, necessitating the continuous development of more effective surveillance strategies and therapeutic interventions to address this ongoing concern.

Although AJCC staging and EBV-DNA quantification are important for prognosis and follow-up in NPC, they are not sufficient for accurately predicting recurrence in all patients. The AJCC/UICC 9th-version staging framework for NPC, introduced for contemporary clinical application in 2025, further refines anatomic risk stratification and emphasizes the continuing need for precise staging in prognostic assessment (8, 9). In parallel, EBV-DNA has evolved from a static pretreatment biomarker into a dynamic circulating tumor DNA indicator. Recent longitudinal EBV-DNA studies, including the EP-SEASON study and the EP-STAR risk-adapted treatment trial, suggest that EBV-DNA clearance patterns during treatment may inform real-time recurrence risk and therapeutic adaptation (10, 11). Nevertheless, EBV-DNA assays are not uniformly available across all centers, and their sensitivity and specificity for individualized prediction can vary according to timing, assay platform, and disease biology (10–12). Therefore, identifying additional, inexpensive, and widely obtainable biomarkers that complement staging and EBV-DNA assessment remains clinically relevant. Inflammatory markers, reflecting the immune response to cancer, have gained attention as potential prognostic tools in various cancers. Systemic inflammation is thought to contribute to tumor growth, metastasis, and immune evasion. Higher levels of immune cells (such as neutrophils, monocytes, and lymphocytes) and inflammatory markers (including C-reactive protein) have been associated with a less favorable prognosis (13, 14). These observations have led to the creation of inflammation-based scores, which incorporate various markers to offer a broader evaluation of a patient's immune status.

One example of such a composite score is the PIV, a metric that combines neutrophil count, platelet count, monocyte count, and lymphocyte count. These elements may reflect the intricate interactions among the immune system, the tumor microenvironment, and systemic inflammation. PIV has been shown to correlate with prognosis in several types of cancer, including non-small cell lung cancer (15) and colorectal cancer (16). However, its potential as a complementary prognostic tool in NPC, particularly for predicting recurrence after chemoradiotherapy alongside conventional staging and EBV-DNA assessment, remains underexplored. Given the critical need for improved recurrence prediction, the role of PIV in NPC warrants investigation. The utility of inflammatory biomarkers, such as PIV, lies in their ability to provide additional host-response information beyond traditional clinical and pathological markers (17, 18). These biomarkers may offer insights into the systemic immune response to the tumor, which could influence tumor behavior and treatment outcomes. Specifically, elevated systemic inflammation, as indicated by a higher PIV, may suggest a more aggressive tumor phenotype, potentially increased metastatic capacity, and a higher risk of recurrence following chemoradiotherapy. Conversely, lower levels of inflammation may be associated with a better response to treatment and a lower risk of recurrence. Given the challenges in accurately predicting recurrence and the need for more refined prognostic tools, this study seeks to assess the possible role of the PIV as an adjunctive biomarker for predicting two-year recurrence in NPC cases after chemoradiotherapy.

## 2 Methods

### 2.1 Participants' enrollment and collecting clinical data

This retrospective investigation involved 306 cases with NPC, all of whom underwent chemoradiotherapy at Xiangyang Central Hospital between February 2017 and April 2023. Patients' follow-up was carried out until either the onset of recurrence or the final assessment in April 2025. Comprehensive baseline clinical and demographic data were retrospectively collected, involving variables such as age, sex, smoking history, alcohol consumption, and body mass index (BMI). Tumor staging, including both T and N stages, was determined on the basis of the 8th edition of the American Joint Committee on Cancer (AJCC) classification for NPC, which was the staging system used during the study period. Because complete retrospective information required for reliable AJCC/UICC 9th-version reclassification, particularly detailed imaging descriptors relevant to updated nodal assessment, was not uniformly available, formal 9th-version restaging was not performed and was considered a limitation of this study. Pretreatment plasma Epstein-Barr virus (EBV) DNA levels, a well-known biomarker for NPC, were documented at baseline within 1 week before treatment initiation. Laboratory parameters, all measured within 1 week prior to the initiation of treatment, consisted of the counts of neutrophils, monocytes, lymphocytes, and platelets, as well as levels of hemoglobin and albumin. The PIV was subsequently derived through a calculation that involves multiplying the neutrophil count, platelet count, and monocyte count, each expressed in units of 10 to the power of 9 per liter, and then dividing this product by the lymphocyte count, also quantified in units of 10 to the power of 9 per liter. Imaging data, primarily from CT and MRI, were precisely reviewed to determine the baseline tumor characteristics, including extent and metastatic involvement. Informed consent and ethical approval were obtained by the Ethics Committee of Xiangyang Central Hospital. The data collection procedures were executed in accordance with the ethical guidelines and standards established by the Declaration of Helsinki.

### 2.2 Inclusion and exclusion criteria

This study included NPC patients who received CCRT at a tertiary medical center between February 2017 and April 2023. The inclusion criteria were: (1) histologically confirmed diagnosis of primitive NPC; (2) treatment with definitive CCRT;(3) a minimum follow-up period of 24 months after treatment completion; and (4) the presence of complete clinical, laboratory, and imaging data was required. The exclusion criteria included: (1) a history of other malignancies; (2) incomplete treatment or discontinuation of chemoradiotherapy due to adverse effects; (3) insufficient follow-up data; and (4) missing baseline or follow-up laboratory data, particularly inflammatory markers or EBV-DNA levels, essential for this study. Based on recurrence status at the 2-year follow-up, stratifying cases into non-recurrent (*n* = 212) and recurrent (*n* = 94) groups was undertaken.

### 2.3 2-year overall survival and follow-up

Patients were monitored for up to 2 years following the completion of CCRT to evaluate recurrence. Recurrence-free survival (RFS) was considered as the period from the end of treatment until the first detection of local, regional, or distant recurrence, or until the last follow-up date for patients who did not experience recurrence. Recurrence was defined as the appearance of local, regional, or distant metastasis within 24 months following the completion of CCRT, as determined by clinical examination, imaging studies (CT or MRI), and/or biopsy confirmation. Out of the 306 NPC patients, 212 were in the non-recurrent group, as they did not experience recurrence during the 2-year follow-up period, while 94 patients, who developed recurrence within 2 years after treatment completion, were placed in the recurrent group. To figure out PIV's prognostic significance, it was attempted to stratify cases into two principal groups on the basis of optimal cut-off value of 381.55, utilizing receiver operating characteristic (ROC) curve analysis. RFS between the two principal groups was compared.

### 2.4 CCRT

All patients received definitive chemoradiotherapy as the primary treatment for NPC. According to the institutional treatment protocol used during the study period, eligible patients first received induction chemotherapy with the GP regimen, consisting of gemcitabine (1,000 mg/m^2^) and cisplatin (80 mg/m^2^), administered every 3 weeks for 3 cycles. After induction chemotherapy, radiotherapy was administered using intensity-modulated radiation therapy (IMRT), with a total dose ranging from 70 to 72 Gy, delivered in daily fractions of 2.0–2.2 Gy, targeting the primary tumor, regional lymph nodes, and areas with potential microscopic disease. During IMRT, concurrent cisplatin-based chemotherapy was given to enhance tumor radiosensitivity, rather than concurrent gemcitabine plus cisplatin. Treatment was generally well tolerated, with adjustments made for individual tolerability, including dose modifications or delays due to toxicities. Patients were monitored for hematologic and gastrointestinal side effects, and regular follow-ups, including blood tests, clinical evaluations, and imaging, were performed to assess treatment response and detect recurrence.

### 2.5 Statistical analysis

Statistical analyses were performed using SPSS 25.0 and R 4.0.5 software. The continuous variables' normality was evaluated through the Shapiro-Wilk test. Data exhibiting a normal distribution were described as mean plus/minus standard deviation (SD), while those that did not adhere to normality were summarized using the median and interquartile range (IQR). Between-group comparisons for normally distributed data were undertaken through the independent samples t-test, while abnormally distributed data underwent analysis using the Mann-Whitney U test. Chi-square test was used for categorical variables to identify distributional differences between groups. We evaluated PIV's prognostic utility for predicting two-year recurrence through ROC curve analysis, using the Youden index to determine the optimal cutoff point. Sensitivity and specificity corresponding to this cutoff point were calculated to describe test performance. Regarding recurrence-free survival (RFS)'s assessment on the basis of the PIV cutoff, we utilized Kaplan-Meier survival analysis, with differences between high and low PIV groups analyzed through the log-rank test. Identification of potential prognostic factors linked to two-year recurrence was implemented via univariate and multivariate Cox proportional hazards regression models. The findings were described as hazard ratios (HR) along with their 95% confidence intervals (CI). Statistical significance was denoted by a two-sided *P* value below 0.05.

## 3 Results

### 3.1 Comparison of baseline characteristics between non-recurrent and recurrent groups of NPC patients after CCRT

Totally, 306 NPC cases who received CCRT were involved, with assigning 212 cases into the non-recurrent group and 94 cases into the recurrent group, as outlined in Table 1. The two principal groups did not exhibit significant differences particularly in baseline characteristics, comprising age (52.44 ± 9.64 vs. 54.35 ± 10.19 years, *P* = 0.118), gender distribution (*P* = 0.298), smoking status (*P* = 0.880), or alcohol consumption (*P* = 0.446). Although an escalated proportion of cases in the non-recurrent group had a BMI surpassing 24 (44.34 vs. 30.85%), this marginal difference did not achieve statistical significance (*P* = 0.065). Furthermore, the levels of Epstein-Barr virus (EBV)-DNA were comparable particularly between the two principal groups (*P* = 0.265). Notably, we detected significant differences particularly in *T* stage distribution (*P* < 0.001). An elevated proportion of cases in the non-recurrent group were classified as T1–T2 (42.92 vs. 20.21%), whereas a heightened proportion of cases in the recurrent group had advanced T3–T4 stages (79.79 vs. 57.08%). Conversely, the absence of significant differences particularly in N stage was noteworthy between the principal groups (*P* = 0.136). Laboratory data uncovered noticeable differences especially in several inflammatory markers between the two principal groups. The recurrent group exhibited a remarkably escalated neutrophil count (4.69 ± 1.02 × 10^9^/L vs. 3.60 ± 0.96 × 10^9^/L, *P* < 0.001), while both monocyte count (0.49 ± 0.14 × 10^9^/L vs. 0.56 ± 0.15 × 10^9^/L, *P* < 0.001) and lymphocyte count (1.78 ± 0.42 × 10^9^/L vs. 1.89 ± 0.40 × 10^9^/L, *P* = 0.027) were remarkably diminished in the recurrent group. The absence of significant differences was noteworthy especially in platelet count (*P* = 0.050), hemoglobin concentration (*P* = 0.518), or serum albumin concentration (*P* = 0.916). The PIV was noticeably elevated in the recurrent group (380.88 ± 204.99 vs. 329.76 ± 193.92, *P* = 0.037), reflecting that elevated PIV may serve as a potential biomarker to forecast NPC's recurrence post-CCRT. These outcomes emphasize the importance of inflammatory profiles and staging parameters in forecasting therapeutic findings particularly in NPC cases.

**Table 1:** Comparison of clinical and laboratory characteristics between the non-recurrent and recurrent groups of nasopharyngeal carcinoma patients after concurrent chemoradiotherapy.

Indices	Non-recurrent group (*n* = 212)	Recurrent group (*n* = 94)	*P* value
Age (years)	52.44 ± 9.64	54.35 ± 10.19	0.118
Sex [*n*(%)]			0.298
Male	144 (67.92)	58 (61.70)	
Female	68 (32.08)	36 (38.30)	
Smoking status [*n*(%)]			0.880
Yes	45 (21.23)	19 (20.21)	
No	167 (78.77)	75 (79.79)	
Alcohol consumption history [*n*(%)]			0.446
Yes	85 (40.09)	33 (35.11)	
No	127 (59.91)	61 (64.89)	
BMI [*n*(%)]			0.065
< 18.5	19 (8.96)	8 (8.51)	
18.5–24	99 (46.70)	57 (60.64)	
≥24	94 (44.34)	29 (30.85)	
EBV-DNA [*n*(%)]			0.265
< 400	121 (57.08)	47 (50.00)	
≥400	91 (42.92)	47 (50.00)	
*T* stage [*n*(%)]			< 0.001
T1–T2	91 (42.92)	19 (20.21)	
T3–T4	121 (57.08)	75 (79.79)	
*N* stage [*n*(%)]			0.136
N0–N1	120 (56.60)	44 (46.81)	
N2–N3	92 (43.40)	50 (53.19)	
Laboratory tests			
Neutrophil ( × 10^9^/L)	3.60 ± 0.96	4.69 ± 1.02	< 0.001
Platelet ( × 10^9^/L)	283.57 ± 48.82	271.66 ± 48.84	0.050
Monocyte ( × 10^9^/L)	0.56 ± 0.15	0.49 ± 0.14	< 0.001
Lymphocyte ( × 10^9^/L)	1.89 ± 0.40	1.78 ± 0.42	0.027
Hb (g/L)	121.03 ± 2.93	120.80 ± 2.93	0.518
Alb (g/L)	42.08 ± 4.00	42.03 ± 3.69	0.916
PIV	329.76 ± 193.92	380.88 ± 204.99	0.037

EBV-DNA, Epstein-Barr virus deoxyribonucleic acid; BMI, body mass index; Hb, hemoglobin; Alb, albumin; PIV, pan-immune-inflammation value.

### 3.2 PIV's predictive value for two-year recurrence of NPC post-CCRT

**Figure 1:**
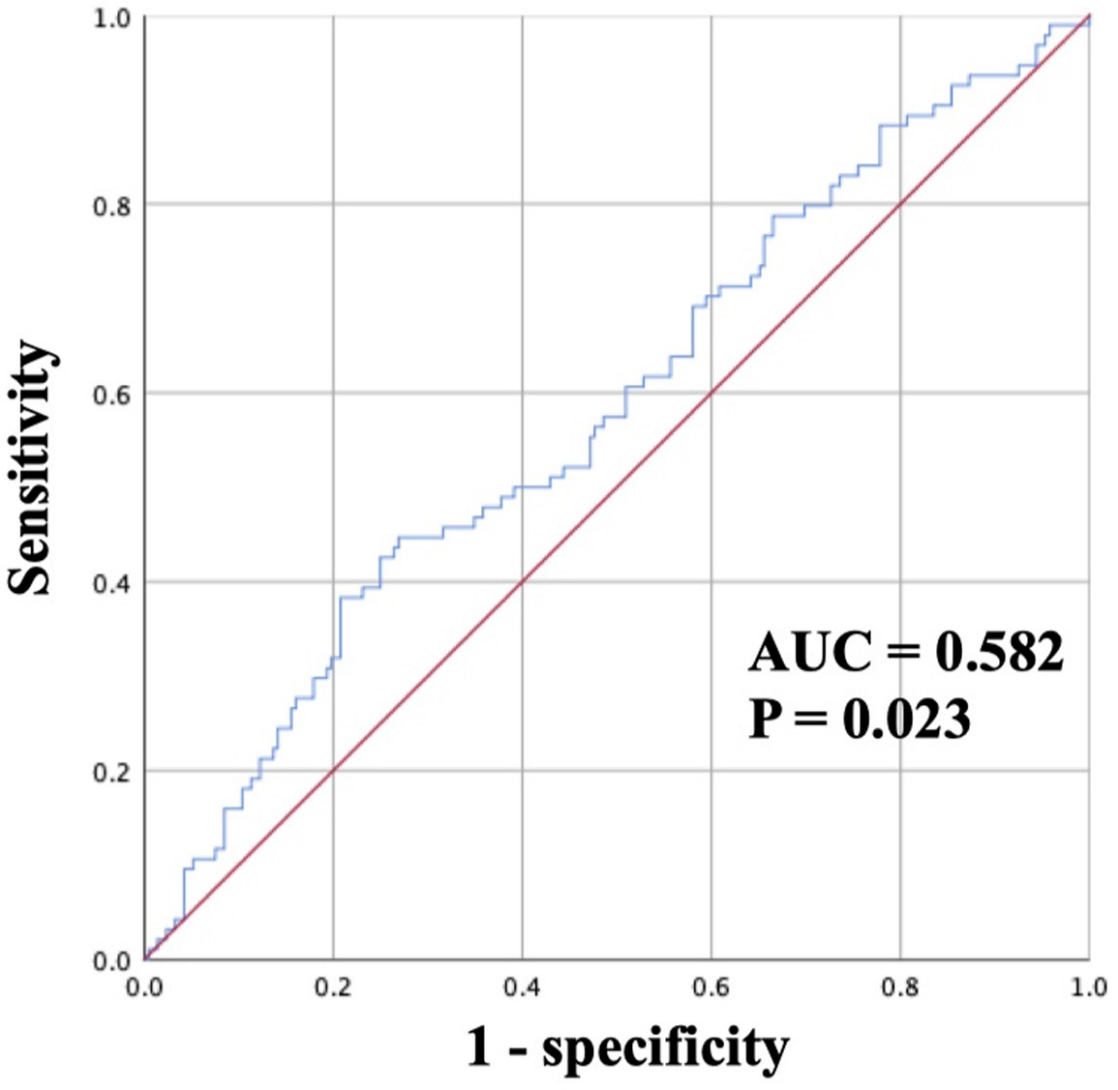
ROC analysis of PIV for predicting 2-year recurrence of nasopharyngeal carcinoma after concurrent chemoradiotherapy. PIV, pan-immune inflammation value. AUC, area under the curve; ROC, receiver operating characteristic.

**Figure 2:**
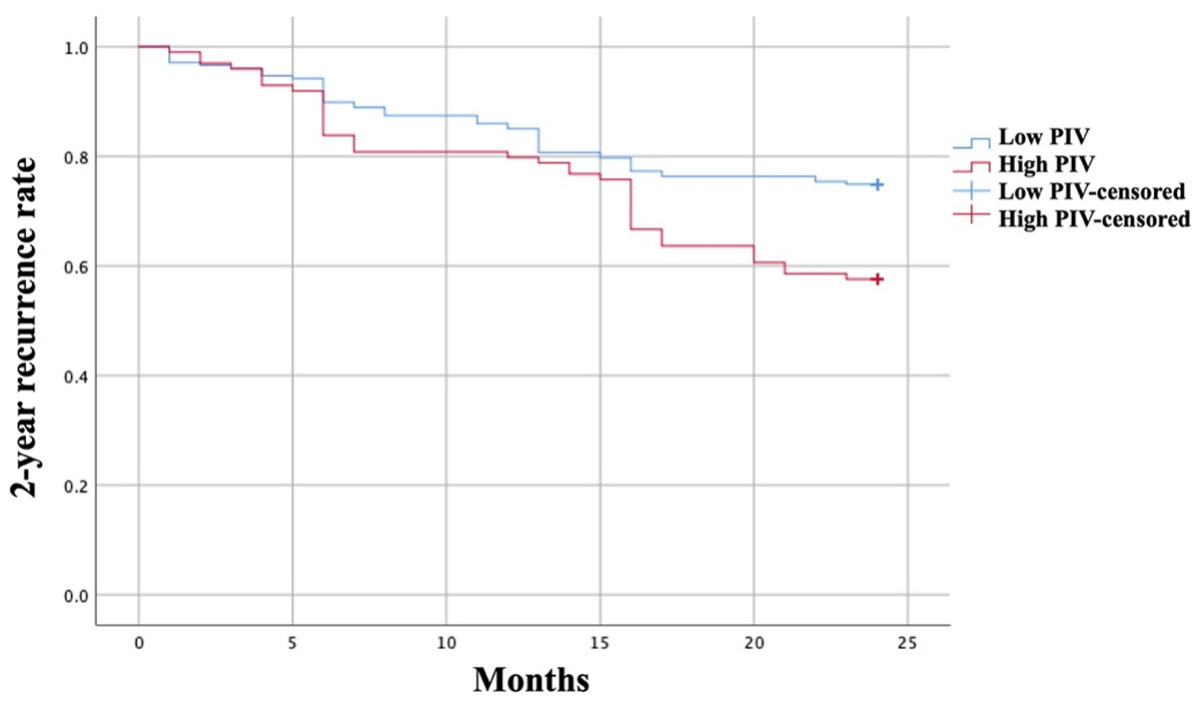
Kaplan-Meier curves for 2-year recurrence rate in nasopharyngeal carcinoma after concurrent chemoradiotherapy. PIV, pan-immune inflammation value.

ROC curve analysis was performed to evaluate PIV's diagnostic performance in forecasting NPC's two-year recurrence after chemoradiotherapy, as outlined in Table 2 and Figure 1. PIV showed modest but statistically significant discriminatory power, with an AUC of 0.582 (95% CI: 0.512–0.651, *P* = 0.023), indicating limited-to-moderate accuracy in distinguishing recurrent cases from non-recurrent ones. The optimal cut-off value for PIV was identified as 381.55, with a sensitivity of 44.7% and a specificity of 73.1%. Given this performance profile, PIV may be more useful as an adjunctive marker for supporting identification of patients at increased recurrence risk than as a standalone tool for excluding recurrence. Kaplan-Meier survival analysis was subsequently implemented to evaluate the impact of PIV on RFS over a 2-year period. Cases were stratified into two groups according to the optimal PIV threshold identified in the ROC analysis: the high PIV group (PIV ≥ 381.55) and the low PIV group (PIV < 381.55). As displayed in the survival curves (Figure 2), the low PIV group exhibited a mean RFS of 20.41 ± 0.48 months (95% CI: 19.48–21.35), while the high PIV group had a mean RFS of 18.69 ± 0.75 months (95% CI: 17.22–20.16). A significant difference in RFS was observed between the two groups according to the log-rank test, with a Chi-square value of 8.418 (*P* = 0.004), suggesting that elevated PIV was associated with shortened recurrence-free survival. These outcomes support the possible utility of PIV as a prognostic marker with modest predictive power for NPC recurrence. Despite its moderate sensitivity, the relatively elevated specificity of PIV suggests possible clinical value as an adjunctive marker, particularly for identifying NPC patients who may require closer post-treatment monitoring after chemoradiotherapy.

**Table 2:** The predictive value of PIV for 2-year recurrence of nasopharyngeal carcinoma after concurrent chemoradiotherapy.

Variables	AUC	95% CI	Best cut-off value	Sensitivity (%)	Specificity (%)	*P* value
PIV	0.582	0.512~0.651	381.55	44.7	73.1	0.023

AUC, area under the curve; CI, confidence interval; PIV, pan-immune-inflammation value.

### 3.3 Cox regression analyses of prognostic factors for two-year recurrence of NPC post-CCRT

After conducting baseline comparisons, variables showing significant differences between the non-recurrent and recurrent groups were further analyzed using univariate Cox regression. As presented in Table 3, *T* stage (HR = 2.450, 95% CI: 1.481–4.054, *P* < 0.001), neutrophil count (HR = 2.203, 95% CI: 1.705–2.400, *P* < 0.001), monocyte count (HR = 0.052, 95% CI: 0.013–0.214, *P* < 0.001), lymphocyte count (HR = 0.606, 95% CI: 0.373–0.987, *P* = 0.044), and PIV (HR = 1.795, 95% CI: 1.195–2.697, *P* = 0.005) were found to be significantly linked with the 2-year recurrence of NPC. Specifically, advanced T stage and elevated neutrophil count were linked to higher recurrence risk, while higher monocyte and lymphocyte counts were protective. Additionally, high PIV correlated with increased recurrence risk, suggesting the role of inflammatory markers in predicting recurrence. To refine these findings and control for confounders, a multivariate Cox regression was implemented. The results, presented in Table 4, showed that *T* stage and PIV remained independent prognostic factors for 2-year recurrence. Although neutrophil, monocyte, and lymphocyte counts showed significance in the univariate analysis, they were excluded from the multivariate model due to their collinearity with PIV. Both T stage (HR = 2.354, 95% CI: 1.421–3.900, *P* = 0.001) and PIV (HR = 1.698, 95% CI: 1.130–2.553, *P* = 0.011) remained significant in the multivariate analysis, further supporting their role in predicting recurrence. These findings highlight the independent prognostic value of *T* stage and PIV in predicting 2-year recurrence of NPC post-CCRT, with both advanced *T* stage and elevated PIV levels being linked to an increased risk of recurrence.

**Table 3:** Univariate Cox regression analysis of prognostic factors for 2-year recurrence of nasopharyngeal carcinoma after concurrent chemoradiotherapy.

Variables	*B*	SE	Wald X2	*P*	HR	95% CI
T stage (T3–T4 vs. T1–T2)	0.896	0.257	12.160	< 0.001	2.450	1.481~4.054
Neutrophils	0.705	0.087	65.361	< 0.001	2.203	1.705~2.400
Monocytes	−2.948	0.718	16.848	< 0.001	0.052	0.013~0.214
Lymphocytes	−0.500	0.248	4.051	0.044	0.606	0.373~0.987
PIV (high vs. low)	0.585	0.208	7.949	0.005	1.795	1.195~2.697

SE, standard error; HR, hazard ratio; CI, confidence interval; PIV, pan-immune-inflammation value.

**Table 4:** Multivariate Cox regression analysis of independent prognostic factors for 2-year recurrence of nasopharyngeal carcinoma after concurrent chemoradiotherapy.

Variables	*B*	SE	Wald X2	*P*	HR	95% CI
T stage (T3–T4 vs. T1–T2)	0.856	0.257	11.056	0.001	2.354	1.421~3.900
PIV (high vs. low)	0.530	0.208	6.480	0.011	1.698	1.130~2.553

SE, standard error; HR, hazard ratio; CI, confidence interval; PIV, pan-immune-inflammation value.

## 4 Discussion

The current investigation suggests that PIV may have prognostic relevance for 2-year recurrence in NPC cases after chemoradiotherapy. Elevated PIV was associated with shorter RFS, and ROC analysis showed statistically significant but modest discriminatory performance. In multivariate Cox regression, advanced *T* stage and elevated PIV remained associated with recurrence risk. These findings indicate that PIV could provide adjunctive prognostic information, although its clinical use should be interpreted cautiously given the retrospective design and moderate sensitivity.

PIV has gained attention because it integrates neutrophil, platelet, monocyte, and lymphocyte counts into a single index reflecting both innate and adaptive immune-inflammatory activity (18–20). Rather than representing a tumor-specific marker, PIV may capture the host inflammatory milieu that can influence tumor progression, treatment response, and residual disease control. Previous studies across malignant and non-malignant diseases have reported associations between elevated PIV and adverse outcomes (21–34); however, these observations should be interpreted as supportive biological context rather than direct evidence for NPC recurrence prediction.

In oncology, inflammation has been associated with tumor initiation, metastasis, treatment resistance, and immune evasion (18, 32–34). Several studies have evaluated PIV in lung, colorectal, gastric, and other cancers, generally suggesting that higher PIV is associated with poorer outcomes (35–41). Mechanistically, elevated neutrophil and platelet counts may contribute to angiogenesis, tumor-cell dissemination, and immune evasion, whereas reduced lymphocyte counts may reflect impaired anti-tumor immunity (42, 43). These mechanisms provide a plausible explanation for why a composite immune-inflammatory index could be associated with recurrence after chemoradiotherapy.

Although the prognostic significance of PIV has been examined in various malignancies, its role in NPC remains less well established. Previous NPC studies have reported associations between high PIV and poorer progression-free survival, overall survival, and distant metastasis-free survival (44–46). Our study extends this literature by focusing specifically on 2-year recurrence after chemoradiotherapy. However, PIV should not be regarded as a replacement for TNM staging or EBV-DNA. Instead, it may provide complementary information reflecting host systemic inflammation, whereas staging captures anatomic tumor burden and EBV-DNA more directly reflects tumor-derived viral DNA.

Chemoradiotherapy remains a central treatment approach for locally advanced NPC, combining systemic therapy with radiation therapy to improve tumor control (6, 47–51). Despite improvements in treatment outcomes, recurrence within the first 2 years remains clinically important. In this context, PIV may help identify a subset of patients with a more unfavorable immune-inflammatory profile. Nevertheless, EBV-DNA has important advantages as a tumor-associated biomarker, particularly when measured longitudinally. Dynamic EBV-DNA or ctDNA clearance during treatment, as shown in recent studies and the EP-STAR trial, can inform real-time risk adaptation and may be more biologically specific to residual tumor burden (10, 11). By contrast, PIV is inexpensive, routinely available, and does not require specialized molecular testing, but it is less tumor-specific and may be influenced by infection, inflammation, nutritional status, and treatment-related changes. Therefore, the most clinically meaningful application may be a combined strategy integrating PIV with AJCC staging and EBV-DNA rather than using PIV alone.

Several limitations should be acknowledged. First, the retrospective single-center design may introduce selection bias, treatment heterogeneity, and follow-up bias, which restrict generalizability. Second, the sensitivity of PIV was modest, indicating that PIV alone is insufficient as a definitive screening or surveillance tool for recurrence. Third, although *T* and *N* stages were recorded according to AJCC 8th edition, complete data for formal AJCC/UICC 9th-version restaging were not uniformly available, limiting our ability to compare prognostic performance between staging editions. Fourth, EBV-DNA was analyzed as a pretreatment baseline variable; longitudinal EBV-DNA clearance during induction chemotherapy, radiotherapy, and post-treatment surveillance was not available for integrated modeling. Finally, although an association between PIV and recurrence was observed, the precise biological mechanisms underlying this linkage remain incompletely defined. Future prospective multicenter studies should validate these findings, incorporate AJCC 9th-version staging and dynamic EBV-DNA monitoring, and evaluate whether combined biomarker models can improve individualized surveillance and treatment adaptation.

## 5 Conclusion

In conclusion, the outcomes suggest that PIV may serve as an adjunctive prognostic biomarker for estimating the risk of 2-year recurrence in NPC cases after chemoradiotherapy. By reflecting systemic inflammatory and immune status, PIV may complement established anatomic staging and EBV-DNA assessment in post-treatment risk stratification. However, further prospective investigations are essential to refine its predictive accuracy, determine its incremental value in combined models, and evaluate its potential role in individualized oncological management.

## Data Availability

The original contributions presented in the study are included in the article/supplementary material, further inquiries can be directed to the corresponding authors.
